# A world without tuberculosis: moving from imagination to reality

**DOI:** 10.1172/JCI162688

**Published:** 2022-09-15

**Authors:** William R. Jacobs

**Affiliations:** Department of Microbiology and Immunology, Albert Einstein College of Medicine, New York, New York, USA.

## Abstract

Tuberculosis (TB), caused by *Mycobacterium tuberculosis* infection, remains a leading cause of death from an infectious agent, resulting in more than a million deaths per year. Despite vaccines and chemotherapies, patients often harbor persister *M. tuberculosis* cells that resist immune assault and chemotherapeutic treatments, resulting in a latent TB infection (LTBI). In this issue of the *JCI*, Sharan et al. used an aerosol-based macaque model to show that weekly treatments with isoniazid and rifapentine for 3 months reduced active *M. tuberculosis* infection and LTBI. Lung tissue from treated animals showed fewer granulomas when compared with the untreated control animals. These findings suggest that it is possible to eliminate persister *M. tuberculosis* cells, thereby eliminating LTBI. If similar elimination routinely occurs in patients undergoing the isoniazid and rifapentine treatment, the hidden reservoir of *M. tuberculosis* associated with LTBI would be greatly reduced, allowing us to imagine, and eventually achieve, a world without TB.

## Tuberculosis has plagued humanity throughout human history

In a 1929 interview, Albert Einstein said, “Imagination is more important than knowledge. Knowledge is limited. Imagination encircles the world” (1). Although Einstein was discussing the theory of relativity, I think his quote is also relevant for research on tuberculosis (TB), a disease that has plagued humanity throughout human history and, sadly, remains 1 of the leading causes of death from an infectious agent, accounting for more than 1 million deaths each year. The associated deaths are particularly saddening as TB is a disease for which we have both a vaccine and sterilizing chemotherapies. I am convinced that TB remains a substantial global health problem due to persisters, the subpopulations of *Mycobacterium tuberculosis* cells in an infection that escape from sterilizing chemotherapies and adaptive immunity (2). To eradicate TB, we need to find effective ways to sterilize the persisters or prevent them from forming. In this issue of the JCI, the report by Sharan et al. provides an incremental, but notable, step toward this goal (3).

Most individuals infected with *M*. *tuberculosis* survive the infection but do not sterilize all infected tissues. As a result, although the TB symptoms improve, the patients harbor persister *M*. *tuberculosis* cells and thus have a latent TB infection (LTBI). These persister organisms can reactivate to active TB later in life. Moreover, these reactivated *M*. *tuberculosis* cells are a source of infectious *M*. *tuberculosis* bacteria that can quickly spread to others through aerosol droplets. Therefore, I imagine TB would be eradicated if we could sterilize LTBI in the human population. Although “imagination encircles the world” is a lofty concept, imagining how to sterilize *M*. *tuberculosis* persisters could affect millions of lives.

## Monitoring *M*. *tuberculosis* infections in a macaque model

The treatment of individuals with LTBI with a 3-month, weekly treatment with isoniazid and rifapentine (3HP) has been previously shown to be efficacious in reducing the rate of TB progression (4). Sharan et al. used the macaque model of aerosol-based *M*. *tuberculosis* infection to show that 3HP is indeed efficacious in these model animals, as has been found in humans (3). Importantly, this manuscript goes beyond just confirming what is known from human clinical studies and provides crucial knowledge using the macaque model that distinguishes this study from human studies.

First, Sharan et al. conducted longitudinal PET/CT and CT analyses of the lungs of infected macaques at planned intervals after defined aerosol infection. By conducting in-depth CT analysis, the researchers showed that 3HP treatment reduced, and sometimes eliminated, granuloma formation. Secondly, Sharan et al. conclusively showed the presence of persistent *M*. *tuberculosis* bacilli in the lungs of macaques that were otherwise free of disease(3). For this purpose, they used simian immunodeficiency virus (SIV), related to human immunodeficiency viruses (HIVs) HIV-1 and HIV-2. In their lab, a model of *M*. *tuberculosis* and SIV coinfection had been developed and shown to lead to a massive depletion of lung CD4^+^ T cells and reactivation of LTBI (5, 6). The authors utilized SIV coinfection strategically — to observe if there were differences in the extent of persistent *M*. *tuberculosis* that remained as a function of 3HP treatment (3). This experiment was warranted because it is challenging to radiologically distinguish between macaques with LTBI and those that have cleared the infection. With their SIV coinfection model, the authors used PET/CT and CT scans to conclusively show that most 3HP-treated macaques had sterilized the infection, while all of the untreated animals had evidence of LTBI reactivation.

Finally, it was possible for Sharan and colleagues to subject the lungs of treated and untreated macaques to CFU analysis at the endpoint to validate the imaging results, whereas it is impossible to carry out such a study in humans (3). Sharan et al. conclusively showed that 3HP treatment reduced the levels of *M*. *tuberculosis* bacilli in the lungs of this cohort of macaques, such that SIV coinfection was unable to result in increased bacillary replication. In contrast, increased *M*. *tuberculosis* levels were observed in the control group. The combined results underscore the power of aerosol TB infection in the macaque model; it is possible to longitudinally evaluate TB disease in the lungs as a function of infection, coinfection, and treatment, but also possible to identify the total lung disease burden at necropsy. While this experiment is important because it validates earlier findings that 3HP treatment is efficacious for LTBI, it also opens the door for testing more chemotherapies and immunotherapies using the LTBI model and the SIV coinfection approach (3).

This study (3) marks an important step toward eradicating TB, as the drug combination kills both the actively growing *M*. *tuberculosis* and the *M*. *tuberculosis* persisters. The *M*. *tuberculosis* macaque model is excellent for the study of LTBI, and advances in PET and CT scanning have provided critical tools with which to follow *M*. *tuberculosis* infections. I firmly believe that it is an inspiring time for TB research. In the last 35 years, we have developed a complete set of tools to manipulate *M*. *tuberculosis* (7). Whole genome sequencing of *M*. *tuberculosis* provided the full complement of genes (8, 9) and paved the way for microarrays to study gene expression of actively growing, dying, or persistent *M*. *tuberculosis* cells (10). Genetics, biochemistry, and X-ray crystallography have allowed for the elucidation of isoniazid action (11). *M*. *tuberculosis* isoniazid persisters can be reproducibly generated in vitro (12) but are eliminated with isoniazid in combination with agents that stimulate respiration (13). Interestingly, isoniazid and rifapentine successfully combined to sterilize *M*. *tuberculosis* cells, including persisters (3). Although a human clinical trial is justified, it is more complicated to demonstrate the sterilization of *M*. *tuberculosis* in humans. If this sterilization can be reproduced in mice, it would provide a cost-effective way to test whether the sterilization is specific to the CDC1551 strain of *M*. *tuberculosis* used in this study (3) or is more broadly applicable. In addition, chemotherapies to further shorten the treatment could be identified. Further testing in genetically modified mouse models could determine whether adaptive or innate immunity plays a role in the sterilization observed with these drug combinations.

## Sharing a vision

Science has come a long way on several fronts, from physics to microbiology. I was so inspired by the images from the James Webb telescope revealing the expansive universe in the past month. I imagined that if Albert Einstein were alive today, he would have been pleased to see proof of his imagination and how much more has yet to be discovered. Since his passing, smallpox has been eradicated, the hepatitis C virus can now be sterilized with drugs in humans, people with HIV live nearly normal everyday lives, and many cures for cancers are being developed. All of these advances came from the imagination and basic research studies conducted by research groups like Sharan et al. (3). I share the vision of the many researchers and health professionals who imagine a world without TB.

## Version 1 09/15/2022

Electronic publication
